# Podocyte hypertrophic stress and detachment precedes hyperglycemia or albuminuria in a rat model of obesity and type2 diabetes-associated nephropathy

**DOI:** 10.1038/s41598-019-54692-z

**Published:** 2019-12-06

**Authors:** Akihiro Minakawa, Akihiro Fukuda, Yuji Sato, Masao Kikuchi, Kazuo Kitamura, Roger C. Wiggins, Shouichi Fujimoto

**Affiliations:** 1 Division of Nephrology, Department of Internal Medicine, Faculty of Medicine, University of Miyazaki, Miyazaki, Japan; 2 Department of Endocrinology, Metabolism, Rheumatology and Nephrology, Faculty of Medicine, Oita University, Yufu, Japan; 3 First Department of Internal Medicine, University of Miyazaki, Miyazaki, Japan; 4 Division of Nephrology, Department of Internal Medicine, University of Michigan, Ann Arbor, Michigan USA; 5 Department of Hemovascular Medicine and Artificial Organs, University of Miyazaki, Miyazaki, Japan

**Keywords:** Prognostic markers, Diabetic nephropathy

## Abstract

Type2 diabetes-associated nephropathy is the commonest cause of renal failure. Mechanisms responsible are controversial. Leptin-deficient hyperphagic Zucker (fa/fa) rats were modeled to test the hypothesis that glomerular enlargement drives podocyte hypertrophic stress leading to accelerated podocyte detachment, podocyte depletion, albuminuria and progression. By 6weeks, prior to development of either hyperglycemia or albuminuria, fa/fa rats were hyperinsulinemic with high urinary IGF1/2 excretion, gaining weight rapidly, and had 1.6-fold greater glomerular volume than controls (P < 0.01). At this time the podocyte number per glomerulus was not yet reduced although podocytes were already hypertrophically stressed as shown by high podocyte phosphor-ribosomal S6 (a marker of mTORC1 activation), high urinary pellet podocin:nephrin mRNA ratio and accelerated podocyte detachment (high urinary pellet podocin:aquaporin2 mRNA ratio). Subsequently, fa/fa rats became both hyperglycemic and albuminuric. 24 hr urine albumin excretion correlated highly with decreasing podocyte density (R^2^ = 0.86), as a consequence of both increasing glomerular volume (R^2^ = 0.70) and decreasing podocyte number (R^2^ = 0.63). Glomerular podocyte loss rate was quantitatively related to podocyte detachment rate measured by urine pellet mRNAs. Glomerulosclerosis occurred when podocyte density reached <50/10^6^um^3^. Reducing food intake by 40% to slow growth reduced podocyte hypertrophic stress and “froze” all elements of the progression process in place, but had small effect on hyperglycemia. Glomerular enlargement caused by high growth factor milieu starting in pre-diabetic kidneys appears to be a primary driver of albuminuria in fa/fa rats and thereby an under-recognized target for progression prevention. Progression risk could be identified prior to onset of hyperglycemia or albuminuria, and monitored non-invasively by urinary pellet podocyte mRNA markers.

## Introduction

Obesity and type2 diabetes mellitus prevalence continues to increase worldwide reflecting population access to high energy food and reduced calorie expenditure necessary to sustain life^1–3^. Diabetes-associated end stage kidney disease (ESKD) is the commonest cause of renal mortality with attendant human and monetary costs^1–4^.

Micro-albuminuria is recognized as an early marker for both obesity and diabetes-associated nephropathy (DAN), with progressive disease being associated with increasing albuminuria, progressive glomerulosclerosis, and accumulating nephron loss eventually culminating in ESKD^5^. Diverse mechanisms contribute to nephron loss including injuries to glomerular, tubule-interstitial and vascular compartments^6,7^.

Normal podocyte density (number per glomerular tuft volume) is necessary to maintain high resolution glomerular filtration through complete coverage of the filtration surface area by foot processes^8^. At the same time podocytes have limited capacity for replacement such that critical reduction of podocyte density (caused by increased glomerular volume and/or reduced podocyte number) is associated with proteinuria, glomerulosclerosis and ESKD in all progressive glomerular diseases so far examined^8–16^.

In type1 and type2 diabetes in man reduced podocyte density is associated with both glomerular enlargement and reduced podocyte number^17–19^. Podocyte injury is present at an early stage and podocyte depletion occurs with advanced DAN^17–19^. Animal model studies implicate a role for mTORC1, the major integrator of growth signaling, in driving podocyte and glomerular injury that reproduces many features associated with progressive DAN in man^20,21^.

We recently reported that increased glomerular volume and impaired podocyte capacity to adapt to hypertrophic stress are both necessary to cause proteinuria and progressive glomerulosclerosis in a non-diabetic rat model^12,15^. Furthermore, reducing the rate of glomerular growth by calorie intake reduction, even when started late and by modest amounts, prevented downstream consequences manifest by podocyte depletion, proteinuria and glomerulosclerosis. Since glomerular enlargement is well-documented in diabetes, and reducing food intake is a therapy available worldwide, we tested the hypothesis that glomerular enlargement could also drive albuminuria and progression in the Zucker rat model of type2 diabetes. In this model homozygous (fa/fa) leptin deficiency causes loss of inhibitory feedback for satiety thereby resulting in primary hyperphagia that drives the cardinal features of type2 diabetes including body weight gain, hyperinsulinemia, peripheral insulin resistance, hyperglycemia and glycosuria. The model also develops progressive albuminuria and glomerulosclerosis, and thereby parallels many aspects of both obesity and type2 DAN in man^22–24^.

## Results

### Time-course

**Figure 1:**
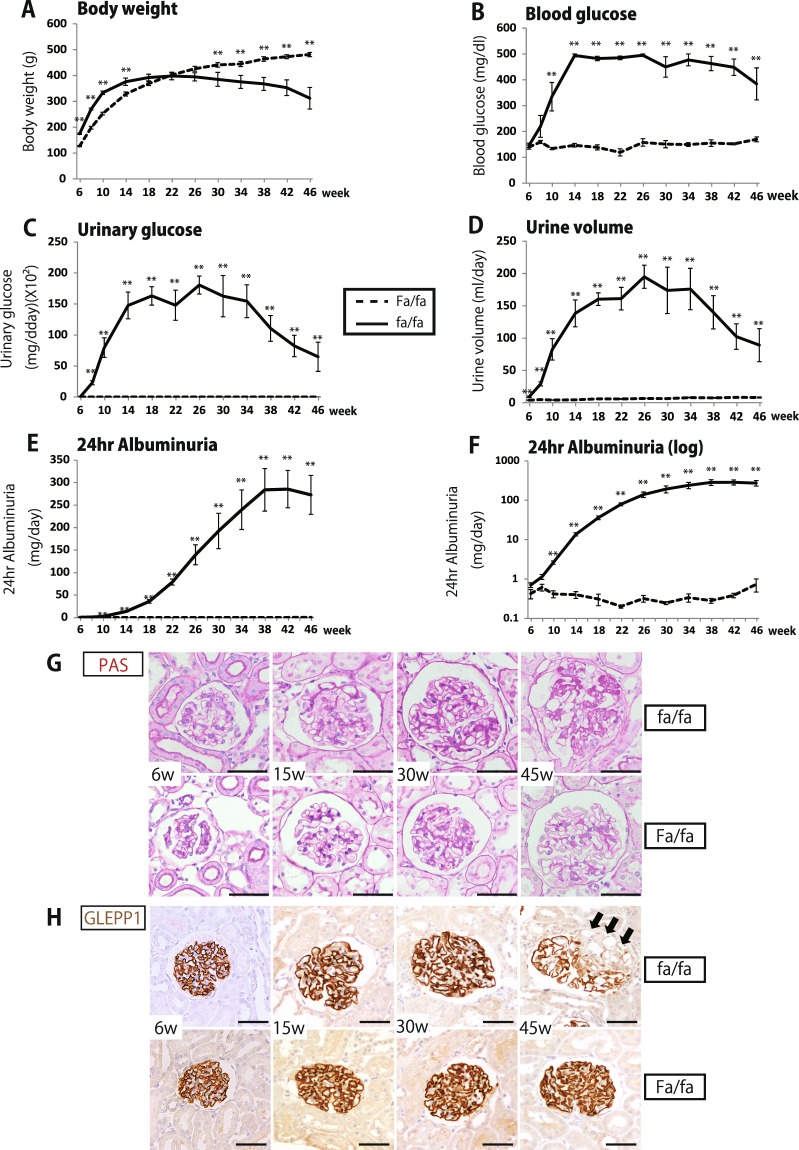
Time course of clinical parameters and histological figures in fa/fa and Fa/fa control rats. (**A**) Body weight gain. (**B**) Blood glucose. (**C**) 24 hr urinary glucose. (**D**) 24 hr urine volume. (**E**) 24 hr urine albumin shown on a linear scale. (**F**) 24 hr urine albumin shown on a log scale. (**G**) Representative PAS staining at 6, 15, 30 and 46 weeks of fa/fa and Fa/fa rats (bar = 50 μm). (**H**) Representative podocytes identified using the podocyte cytoplasmic marker glomerular epithelial protein 1 (GLEPP1) by immune-peroxidase (bar = 50 μm). Glomeruli appear larger in fa/fa rats than Fa/fa rats with increasing mesangial expansion over time in fa/fa rats shown by PAS staining. By 46weeks segmentally sclerotic lesions were present in fa/fa rats affecting 47 ± 28% of glomeruli, but were absent from glomeruli of Fa/fa rats. Data shown as the mean ± SEM. Statistically significant differences are shown by asterisks (*P < 0.05, **P < 0.01).

Homozygous fa/fa rats were already significantly heavier than Fa/fa controls by 6weeks of age and continued to gain weight faster than control rats prior to onset of overt hyperglycemia (Fig. 1A). In association with persistent high level glycosuria and by 18weeks fa/fa rat weight had plateaued and by 30weeks body weight had decreased to below that of Fa/fa control which continued weight gain through 46weeks of observation. By 10weeks fa/fa rats had developed overt hyperglycemia (Fig. 1B) associated with glycosuria (Fig. 1C), and increased urine volume due to glycosuria-associated osmotic diuresis (Fig. 1D). 24 hr urine albumin excretion in fa/fa rats became significantly increased by 10weeks, continued to increase to high levels by 38weeks and plateaued thereafter (Fig. 1E,F). Significant albuminuria did not develop in Fa/fa control rats. Blood pressure measured by tail cuff remained in the normal range (systolic 110–130 mmHg) with no significant difference between fa/fa and Fa/fa controls at any time point (data not shown).

### Histologic changes and morphometry

**Figure 2:**
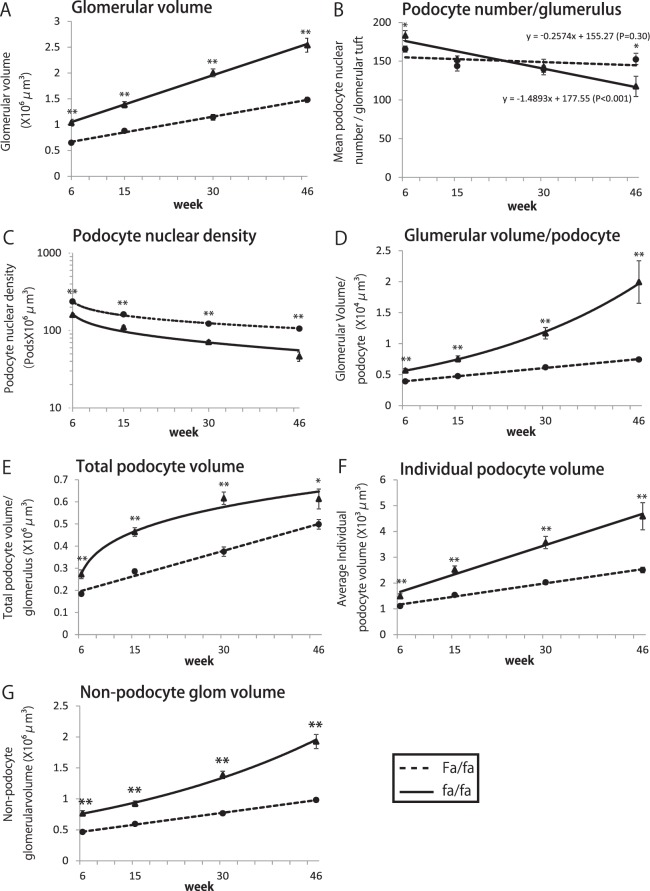
Morphometric parameters in fa/fa and fa/fa control rats. (**A**) Glomerular volume. (**B**) Podocyte nuclear number per glomerulus. (**C**) Podocyte nuclear density. (**D**) Glomerular volume per podocyte (**E**) Total podocyte volume per glomerulus (**F**) Average individual podocyte volume (**G**) Non-podocyte glomerular volume. By 6weeks (pre-diabetic) glomerular volume in fa/fa rats was significantly increased above that of Fa/fa rats (P < 0.01). Over time podocyte number per glomerulus in fa/fa rats decreased at a rate of −1.49 podocytes per week (P < 0.01), while podocyte number per glomerulus did not change significantly over time in Fa/fa rats (P = 0.30). At 6weeks the podocyte nuclear density (number density) was significantly lower in fa/fa rats than Fa/fa controls (<0.01). To compensate for reduced podocyte density the average individual podocyte volume was increased 1.3-fold in fa/fa rats above control by 6weeks (P < 0.01). However, at 6weeks total podocyte volume (podocyte number x size) had not fully adapted to glomerular volume increase as shown by the glomerular volume per podocyte. Statistically significant differences are shown by asterisks (*P < 0.05, **P < 0.01).

Fig. 1G,H, Supplemental Fig. 1A,B and Fig. 2 shows representative histological findings and morphometry at 6, 15, 30 and 46weeks in fa/fa and Fa/fa controls. Glomeruli of fa/fa rats appeared larger than those of Fa/fa rats at each time point. As shown in Fig. 2A, by 6weeks (prior to development of overt diabetes or albuminuria) glomerular volume of fa/fa rats was already 1.6-fold greater than glomerular volume of Fa/fa rats (P < 0.01). The podocyte number per glomerulus in fa/fa rat glomeruli was slightly but significantly higher than in control littermates at 6weeks (P = 0.04) (Fig. 2B). Podocyte number per glomerulus decreased throughout the time course in fa/fa rats with a slope of −1.49/week (P < 0.01), but did not decrease significantly in Fa/fa controls. Podocyte density (number per volume) in fa/fa rats was decreased compared to Fa/fa controls by 6weeks (P < 0.01) and continued to decrease so that by 46weeks the podocyte density had reached low levels of 50 × 10^6^/um^3^ associated with development of glomerulosclerosis (Fig. 2C). Glomerular volume per podocyte in fa/fa rats was increased compared to Fa/fa controls by 6weeks (P < 0.01) and continued to increase over the 46week observation period (Fig. 2D). Total podocyte cell volume per glomerulus and average individual podocyte cell volume were already 1.5-fold and 1.3-fold respectively above control at 6weeks (P < 0.01) and became increasingly different from control over the 46week observation period (Fig. 2E,F). In fa/fa rats by 30weeks PAS-positive expanded mesangial matrix was present (Fig. 1G) as also indicated by the increasing non-podocyte glomerular volume (Fig. 2G). By 46weeks expanded PAS-positive mesangial matrix was present in glomeruli (Fig. 1G) in association with segmental sclerosis lesions in 47 ± 28% of glomeruli of fa/fa as assessed by Glepp1 peroxidase and AZAN staining (Fig. 1H and Supplemental Fig. 1A).

### Relationship of morphometric parameters to albuminuria

**Figure 3:**
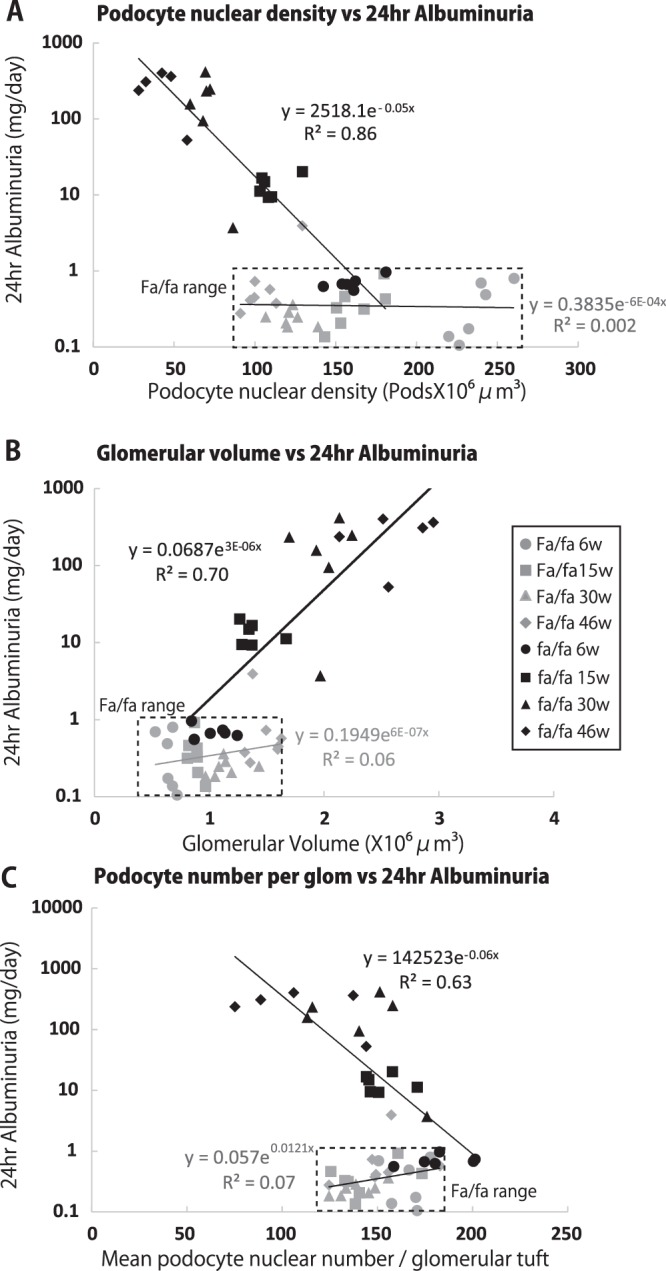
Relationship of morphometric parameters to 24 hr urine albumin excretion. (**A**) Relationship between podocyte nuclear density and 24 hr urine albumin excretion. (**B**) Relationship between glomerular volume and 24 hr urine albumin excretion. (**C**) Relationship between podocyte nuclear number per glomerulus to 24 hr urine albumin excretion. There is a very strong linear relationship between podocyte nuclear density and 24 hr albuminuria for fa/fa rats (R^2^ = 0.86, P < 0.01) that was not present for Fa/fa rats. The relationship of 24 hr urine albumin to glomerular volume showed an R^2^ = 0.70 (P < 0.01). Similarly, there was a strong relationship between 24 hr urine albumin and podocyte number per glomerulus (R^2^ = 0.63, P < 0.01). Panel A shows that at a podocyte density of about 100pods/10^6^um^3^ fa/fa rats had a 24 hr urine albumin of about 10 mg/day. In contrast at the same podocyte density Fa/fa rat 24 hr urine albumin was <1 mg/day. Therefore fa/fa rat podocytes had increased susceptibility to hypertrophic stress at the same podocyte density.

24 hr urine albumin excretion was plotted against glomerular morphometric parameters (Fig. 3). There was a very strong relationship between podocyte nuclear density and 24 hr albuminuria (R^2^ = 0.86, P < 0.01) (Fig. 3A). Since podocyte density is the ratio of glomerular volume to podocyte number per glomerulus these parameters were separately plotted (Fig. 3B,C). The relationship of 24 hr urine albumin to glomerular volume showed an R^2^ = 0.70 (P < 0.01). Similarly, there was a strong relationship between 24 hr urine albumin and podocyte number per glomerulus (R^2^ = 0.63, P < 0.01). Thus decreased podocyte density caused initially by increased glomerular volume alone, and subsequently by a combination of decreasing podocyte number compounded by increasing glomerular volume is strongly associated with albuminuria. However, fa/fa rat podocytes also appeared to be more susceptible than Fa/fa podocytes to hypertrophic stress as shown by the higher level of albuminuria at a density of 100 podocytes/10^6^um^3^ (Fig. 3A), implying that a podocyte susceptibility factor was also required for albuminuria to be present.

### High growth milieu and podocyte hypertrophic stress

**Figure 4:**
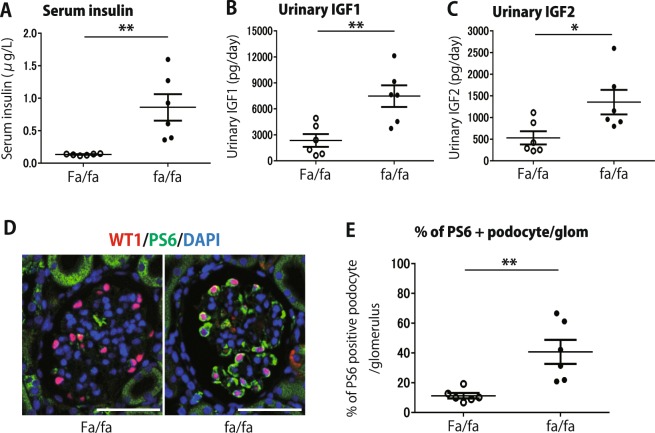
Pre-diabetic 6week values for serum insulin, 24 hr urine growth factor excretion and phosph-S6 immunostaining of Fa/fa and fa/fa rats. (**A**) Serum insulin at 6week. (**B**) 24 hr urinary IGF1 at 6week. (**C**) 24 hr urinary IGF2 at 6week. (**D**) Immunofluorescent identification of phosphorylated ribosomal S6 (green cytoplasmic staining) at 6weeks prior to onset of overt hyperglycemia. Podocytes are shown by red WT1 nuclear staining. DAPI (blue) shows all nuclei. (**E**) Quantitation of glomerular ribosomal phosphor-S6 kinase in fa/fa versus Fa/fa rat glomeruli at 6weeks. These data show the high growth factor milieu prior to onset of overt diabetes in fa/fa rats compared to Fa/fa controls and increased ribosomal-S6 phosphorylation as a result of mTORC1 activation. *n* = 6 for the Fa/fa group, *n* = 6 for the fa/fa group, *P < 0.05 and **P < 0.01, assessed using the Mann-Whitney U test.

Fig. 4A–C show that at 6weeks, prior to onset of glycosuria or albuminuria, blood insulin levels were 6.4-fold higher in fa/fa rats than in Fa/fa controls, and IGF1 and IGF2 (measured as 24 hr urine excretion) were 3.2-fold and 2.1-fold increased respectively. These data reflect an increased growth milieu in fa/fa kidneys. At this time, glomerular volume and average podocyte volume in fa/fa rats were already 1.6-fold and 1.3-fold above control respectively (Fig. 2A,F). Furthermore, the ratio of glomerular volume to total podocyte volume was higher in fa/fa than in Fa/fa controls (P < 0.01) (Fig. 2D) demonstrating that podocyte hypertrophy had not kept pace with glomerular volume increase. Growth factors and nutrients drive cell growth via the mTORC1 pathway in part by phosphorylation of ribosomal protein S6. As shown in Fig. 4D,E, by 6weeks podocyte ribosomal S6 phosphorylation was already significantly increased in fa/fa rat versus controls, compatible with hypertrophic podocyte stress being already present by 6weeks.

### Urinary mRNA markers of podocyte hypertrophic stress

**Figure 5:**
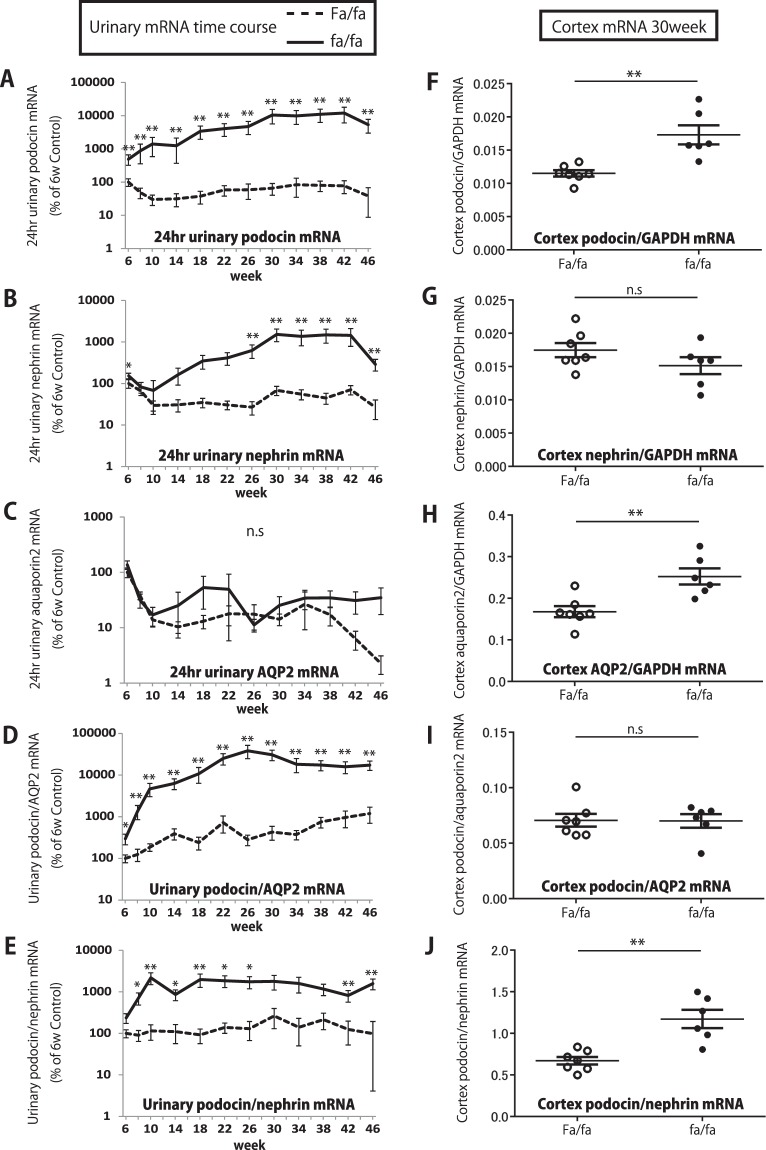
Time course of urinary pellet and kidney cortex mRNAs in fa/fa and Fa/fa control rats. (**A**) 24 hr urinary podocin mRNA. (**B**) 24 hr urinary nephrin mRNA. (**C**) 24 hr urinary aquaporin 2 mRNA. (**D**) Urinary podocin:aquaporin 2 mRNA ratio per day. (**E**) Urinary podocin:nephrin mRNA ratio per day. (**F**) Kidney cortex podocin:GAPDH mRNA ratio at 30weeks. (**G**) Kidney cortex nephrin:GAPDH mRNA ratio at 30weeks. (**H**) Kidney cortex aquaporin 2:GAPDH mRNA ratio at 30weeks. (**I**) Kidney cortex podocin:aquaporin 2 mRNA ratio at 30weeks. (**J**) Kidney cortex podocin:nephrin mRNA ratio at 30weeks. By 6weeks, prior to onset of overt diabetes or albuminuria, the urinary podocin mRNA excretion and the podocin:aquaporin2 mRNA ratio were already significantly increased above Fa/fa control (P=0.04) and increased over the time course. The urinary podocin:nephrin mRNA ratio in the urine pellet as a measure of podocyte hypertrophic stress was also decreased by 8weeks (P < 0.05). For comparison at 30weeks the renal cortical podocin:GAPDH mRNA ratio in fa/fa rats was 1.5-fold increased (P < 0.01) Statistically significant differences are shown by asterisks (*P < 0.05, **P < 0.01).

Hypertrophic podocyte stress causes accelerated podocyte detachment which can be monitored non-invasively by measuring podocyte mRNAs in the urine pellet^12,15^. Figure 5A–D shows 24 hr urinary pellet mRNAs for two podocyte markers (NPHS2/podocin and NPHS1/nephrin) and a distal tubular/collecting duct marker (aquaporin2). Also shown is the podocin:aquaporin2 mRNA ratio as a measure of the relative rate of podocin mRNA excretion in relation to a tubular mRNA marker (aquaporin2). Figure 5C confirms that 24 hr urinary pellet aquaporin2 mRNA levels were comparable between fa/fa and Fa/fa rats over the time-course in spite of large differences in urine volume between fa/fa and Fa/fa rats. By 6weeks, prior to onset of hyperglycemia or albuminuria, both the urinary pellet podocin mRNA and podocin:aquaporin2 mRNA ratio in fa/fa rat were already significantly increased above that of controls (P = 0.04), compatible with hypertrophic podocyte stress causing accelerated podocyte detachment at this early time point. Urinary podocin mRNA and podocin:aquaporin2 mRNA ratio subsequently increased reaching a plateau by 22weeks and persisted throughout the 46weeks of study at an average 88-fold higher levels in fa/fa rat urine compared to Fa/fa rat controls over the period of observation. The relative level of two podocyte-specific mRNAs (podocin and nephrin) is a non-invasive measure of podocyte stress caused by relative down-regulation of nephrin versus podocin^10,25^. Figure 5E shows that by 8weeks, prior to development of hyperglycemia or albuminuria, the urinary podocin:nephrin mRNA ratio was already significantly increased above control (P < 0.01). Figure 5F–J show that the changes observed in the urinary pellet were also present in renal cortex at the 30week time point.

The high nutrient and growth factor milieu in fa/fa rats might be expected to facilitate accelerated cell cycling as a part of the growth process. To assess this possibility, we used Ki67 as a cell cycle marker and WT1 and PAX8 as podocyte and parietal epithelial cell (PEC) markers respectively. As shown in Supplemental Table 1 although Ki67 labelling was present in glomeruli, interstitial and tubular compartments in both fa/fa and Fa/fa rats, there was no statistically significant difference between these groups. Furthermore, we were not able to identify double-label WT1/Ki67 positive cells (or double label PAX8/Ki67 PECs) in fa/fa rat kidney although double labelled cells were detectable in 2day old kidneys (Supplemental Fig. 2), suggesting that if de novo podocyte or PEC replenishment was occurring it was below the threshold of detection.

### Food intake reduction (CR) “freezes” the progression process in place

**Figure 6:**
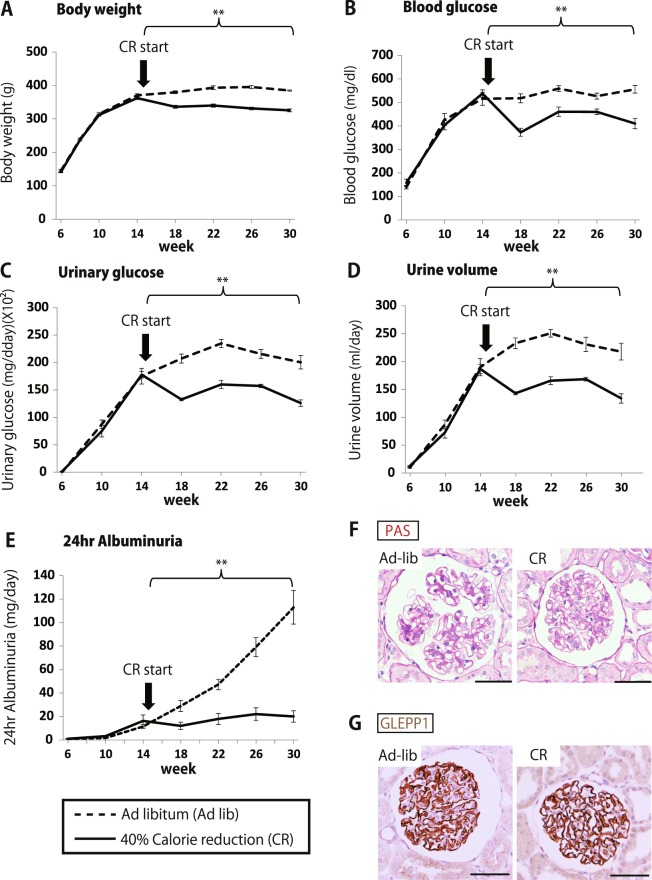
Time course of effect of switching to a calorie-reduced diet at 15weeks and histological figures in the fa/fa rat. (**A**) Body weight. (**B**) Blood glucose. (**C**) 24 hr urine glucose excretion. (**D**) Urine volume. (**E**) 24 hr urinary albumin excretion. (**F**) Representative PAS staining at 30 weeks of ad-libitum and 40% calorie intake reduction (CR) group in fa/fa rats (bar = 50 μm). (**G**) Representative podocytes identified using the podocyte cytoplasmic marker glomerular epithelial protein 1 (GLEPP1) by immune-peroxidase at 30 weeks of ad-libitum and 40% calorie intake reduction (CR) group in fa/fa rats (bar = 50 μm). The 40% reduced food intake group (CR, solid line, n = 6) gained less weight, had lower blood glucose, less glycosuria, lower urine volume and less albuminuria compared with the ad libitum diet group (dashed line, n = 5). No histologic differences were detected between CR and ad libitum fed rats. **P < 0.01, assessed using the Mann-Whitney U test at the average 15–30week.

**Figure 7:**
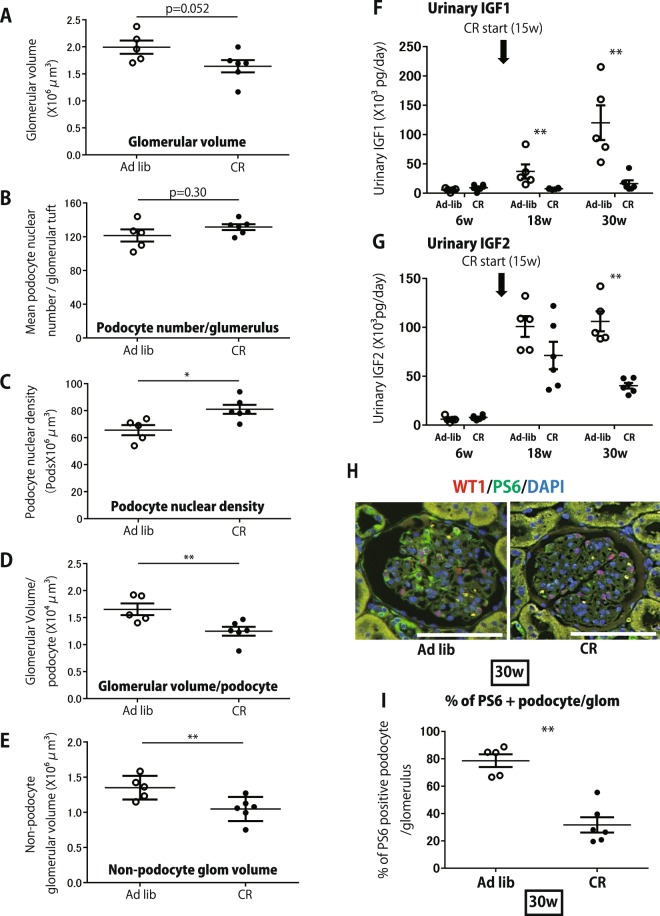
Morphometric analysis and effect on reduced calorie intake (40%CR) values for 24 hr urine growth factor excretion and phosph-S6 immunostaining of fa/fa rats. (**A**) Glomerular volume. (**B**) Mean podocyte nuclear number per glomerular tuft. (**C**) Podocyte nuclear density. (**D**) Glomerular volume per podocyte. (**E**) Non-podocyte glomerular volume. (**F**) Effect of 40% CR on 24 hr urinary IGF1. (**G**) Effect of 40% CR on 24 hr urinary IGF2. (**H**) Representative immune-histologic findings at 30 weeks of ad-libitum and 40%CR fa/fa rats. Phosphorylation of ribosomal S6 in glomeruli (green immunofluorescence). Podocyte nuclei are shown by red WT1 immunofluorescent staining. DAPI is shown in blue. (bar = 100 μm). (**I**) Quantification of % of ribosomal phosphor-S6 positive podocytes per glomerulus. The CR rats had less increase in glomerular volume although podocyte number per glomerulus did not change significantly, resulting in lower podocyte density reduction in CR rats than occurred in the ad libitum-fed group. CR was associated with lower urinary IGF1/2 excretion and reduced mTORC1-induced hypertrophic podocyte stress. n = 5 for ad-libitum diet group, n = 6 for 40%CR group, *P < 0.05 and **P < 0.01, assessed using the Mann-Whitney U test.

If excess nutrient intake causes the altered kidney milieu that in turn causes podocyte hypertrophic stress and accelerated detachment leading to proteinuria and glomerulosclerolsis, then reducing nutrient intake should halt this process. To test this hypothesis rats were allowed to develop diabetes up to 15weeks on an ad-libitum diet and were then placed on a 40% reduced food intake (CR) diet in comparison to an ad-libitum fed group. As shown in Fig. 6 and Supplemental Fig. 3, CR had the effect of stabilizing (“freezing”) all parameters at the level they were at when CR was initiated at 15weeks of age. Thus, although CR rats had statistically significantly less weight gain and lower blood glucose levels, hyperglycemia persisted at high levels over the 15weeks of observation (from week15 to week30). Ad libitum fed fa/fa rat 24 hr urinary albumin excretion continued to increase to 100 mg/day, while CR rats maintained stable albuminuria at about 20 mg/day. Histology showed increased PAS-positive mesangial matrix in ad-libitum fed but not CR rats (Fig. 6F,G). Morphometry (Fig. 7A–E) showed that CR rats had lower glomerular volume (P = 0.05), lower glomerular volume per podocyte and non-podocyte glomerular volume (P < 0.01) and a higher podocyte nuclear density (P < 0.05), although podocyte number per glomerulus was not significantly different (P = 0.30). Thus CR rats at 30weeks had similar morphometric parameters to the ad-libitum fed rats at 15weeks (see Fig. 2), consistent with CR slowing or halting growth-induced processes.

Figure 7C shows that the effect of CR was to stabilize podocyte density at a level of 82 podocytes/10^6^um^3^ which, from Fig. 3A, would be predicted to correspond to a 24 hr urinary albumin excretion of about 20 mg/day similar to that observed in Fig. 6E. In contrast, the ad-libitum fed rat podocyte nuclear density decreased to 65/10^6^um^3^ primarily as a result of continued glomerular growth. From Fig. 3A a podocyte density of 65/10^6^um^3^ is predicted to correspond to a 24 hr urinary albumin excretion rate of close to 100 mg/day as observed in Fig. 6E. Thus the observed 24 hr urinary albumin excretion values correspond closely to the values predicted by the podocyte density measurements thereby providing additional support for the concept that podocyte density is the major determinant of albuminuria.

### Effect of CR on the growth milieu and podocyte hypertrophic stress

Fig. 7F–I shows that CR was associated with lower urinary IGF1/2 excretion. Furthermore, after 15weeks of CR glomerular ribosomal phosphor-S6 positive podocytes were reduced from 78% to 28% (P < 0.01) demonstrating that CR also reduced mTORC1-induced hypertrophic podocyte stress.

### Reduced podocyte stress on the CR diet could be monitored by reduced urine podocyte mRNA markers

**Figure 8:**
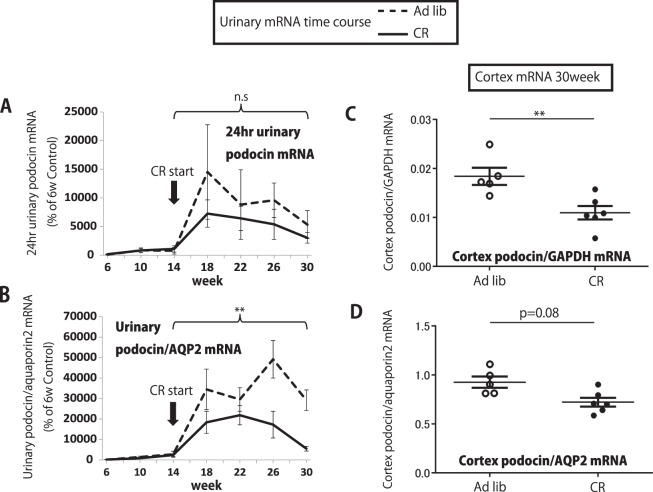
Urinary and kidney cortex mRNA in ad-libitum and calorie intake reduction (CR) groups in fa/fa rats. (**A**) Time course of 24 hr urinary podocin mRNA. (**B**) Time course of urinary podocin:aquaporin2 mRNA ratio. (**C**) Kidney cortex podocin:GAPDH mRNA ratio at 30weeks. (**D**) Kidney cortex podocin:aquaporin 2 mRNA ratio at 30weeks. CR was associated with lower urinary pellet and renal cortical podocyte mRNA. n = 5 for ad-libitum diet group, n = 6 for 40% calorie intake reduction group, *P < 0.05 and **P < 0.01, assessed using the Mann-Whitney U test using the average value of urinary data over the 15–30week time period.

Figure 8 shows that CR was associated with a reduced rate of podocyte detachment as measured in both urinary pellet and in renal cortex (P < 0.01). Although the cortical podocin:nephrin mRNA ratio was reduced by CR (P < 0.05), it was not reduced in the urinary pellet (Supplemental Fig. 3), suggesting that detaching podocytes were those that were stressed and had a high urinary podocin:nephrin mRNA ratio.

## Discussion

Insulin and insulin-like growth factor (IGF-1/2) signaling (IIS pathway) in mammals, characteristically activated by nutrients and inhibited by starvation, is the paralogue of the insulin-like receptor *daf*-2 in the nematode C. *elegans* that has provided remarkable insights into relationships between nutrition, growth and longevity^26–28^. IGF-1 under the influence of both growth hormone and local factors is a major driver of kidney growth^29^. Cell growth is coordinated through mTORC1 activation driven both by growth factors binding to cell surface receptors and nutrient sensing of amino acids and glucose^30,31^. Hyperphagic fa/fa rats with secondary hyperinsulinemia, high IGF1/2, and large nutrient intake are therefore predisposed to activate mTORC1 and thereby to grow rapidly, including rapid glomerular growth. At the same time the podocyte is a structurally complex cell with limited capacity to divide or hypertrophy rapidly. Furthermore, primary dysregulation of the mTORC1 pathway in podocytes is proven to cause thickened GBM, proteinuria and other diabetes-like pathologic changes in the absence of hyperglycemia^12,15,20,21,32^. Thus, in fa/fa rats a high growth milieu can drive both glomerular volume enlargement and podocyte mTORC1 activation. In this setting the stressor (glomerular enlargement) and the susceptibility factor (podocyte mTORC1 activation) combine to cause accelerated podocyte detachment, albuminuria, glomerulosclerosis, and ultimately progression to ESKD, as previously reported for a non-diabetic model^12,15^.

Kriz and Lemley have previously emphasized that podocyte loss from glomeruli occurs primarily by detachment^33^. We therefore evaluated whether podocyte mRNA markers detected in the urinary pellet could quantitatively account for the number of podocytes lost from fa/fa rat glomeruli over the 40week period of observation. As shown in Supplemental Table 2, we estimate that >70% of podocytes lost from glomeruli could in fact be accounted for in the urinary pellet. Non-invasive markers that quantitatively reflect podocyte stress prior to hyperglycemia or albuminuria onset and that non-invasively report the rate of podocyte detachment from glomeruli have potentially clinical utility. We have previously reported similar associations in other models^10–12,15^ as well as in human Alport syndrome^14,34^, IgA nephropathy^35^, anti-GBM disease^16^ and allograft failure^36^. Other investigators have reported similar findings^37–42^.

By 8–10weeks in homozygous fa/fa rats overt diabetes reflected by hyperglycemia, glycosuria and polyuria was present. Increased albumin excretion was also present by 10weeks, and reached high levels by 38weeks. At first sight albuminuria might seem attributable to hyperglycemia itself, however, decreasing podocyte density is an alternative explanation for the progressive increase in albuminuria. This is an important distinction because if proteinuria and progressive glomerulosclerosis are significantly caused by glomerular volume enlargement this would identify an under-recognized therapeutic target for preventing progression. If glomerular enlargement causing podocyte hypertrophic stress is indeed a major driver of albuminuria in the fa/fa rat then one would expect that there would be a direct relationship between the 24 hr urinary albumin excretion and glomerular volume itself, as was observed (R^2^ = 0.70, P < 0.001). Furthermore, if the effect of glomerular volume enlargement in causing albuminuria was through podocyte hypertrophic stress then one would expect that podocyte density (glomerular volume in relation to podocyte number) would show an even stronger relationship with albuminuria than did glomerular volume alone, as was observed (R^2^ = 0.86, P < 0.001). Glomerulosclerosis was present in fa/fa rat glomeruli by 46weeks when podocyte density had reached low levels (50/10^6^um^3^) as a result of the combination of glomerular volume enlargement and podocyte depletion. Podocyte density at this level is associated with glomerulosclerosis in all models and human glomerular diseases so far examined^12–19^. These data are therefore compatible with prior work showing that glomerular volume increase *per se* in the setting of podocyte susceptibility to hypertrophic stress drives albuminuria and glomerulosclerosis^12–16^.

Further support for this concept is provided by the food reduction (CR) study initiated at 15weeks after hyperglycemia and albuminuria were already established. In spite of persistent hyperglycemia, CR essentially “froze” the progression process in place such that all parameters including urinary IGF1/2, glomerular enlargement, podocyte density reduction, podocyte mTORC1 activation (ribosomal phosphor-S6), podocyte detachment rate, urinary podocyte markers and albuminuria remained at the stage they were at when CR was initiated. This suggests that podocytes were able to adapt to a lower stress level over the 15weeks of CR in spite of persistent hyperglycemia. Therefore, in the fa/fa rat hyperglycemia *per se* did not appear to be either necessary to initiate progression nor sufficient to drive the progression process once established.

**Figure 9:**
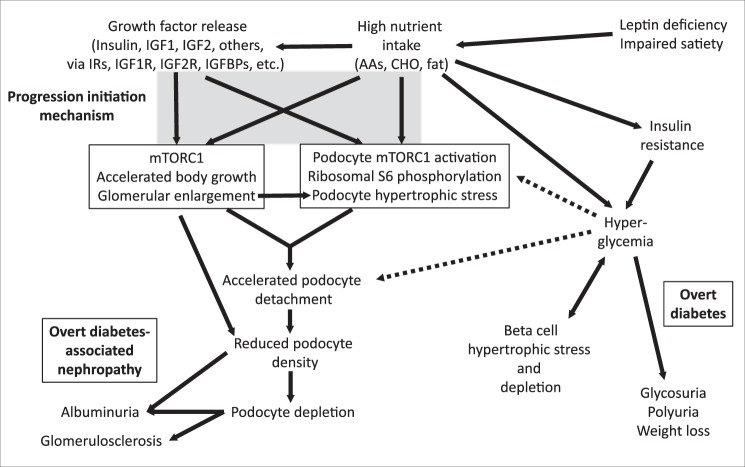
Schematic hypothesis for growth-induced progression derived from the fa/fa model. Nutrition-induced insulin and insulin-like growth factors (IGF-1/2) activate their individual receptors (insulin receptors [IRA and IRB], IGF-1R and IGF-2R, and their hybrid receptors comprising combinations of IR and IGF-1R proteins^53^. IGF-1 availability and local function is regulated through 6 different IGF1 binding proteins (IGFBPs1–6). Cell growth occurs through GF-induced activation of the mTORC1 complex which simultaneously senses nutrient availability including amino acids (leucine, arginine) and glucose^30,31,54^. In fa/fa rats high level nutrient intake drives high growth factor expression from birth resulting accelerated body and organ growth. IGF-1 promotes both glomerular volume enlargement and hyperfiltration^29,55–57^. Glomerular enlargement requires podocytes to hypertrophy to cover the expanding filtration surface with foot processes through increased protein synthesis via mTORC1 kinase-induced phosphorylation/activation of S6 kinase which in turn phosphorylates ribosomal S6. Thus, the collaboration between growth factors, nutrients, glomerular volume and podocytes represents the core components of a “progression initiation mechanism” (see the shaded box). Podocyte hypertrophic stress is represented in this report by the triad of high level podocyte ribosomal S6 phosphorylation, relative down-regulation of the podocyte-specific transcript nephrin versus podocin, and accelerated podocyte detachment into the filtrate. Glomerular volume increase and accelerated podocyte detachment both drive reduced podocyte density. The level of reduced podocyte density in concert with podocyte stress determines amount of albumin leak through the filter, and thereby degree of albuminuria. Mesangial expansion reduces the filtration area required for foot process coverage to preserve filter integrity, but when podocyte density reduction reaches critically low values fibrosis supervenes at sites of podocyte depletion. In parallel, pancreatic islet beta cells hypertrophy to increase insulin release to adapt to increasing insulin resistance, but eventually fail and become depleted in association with loss of blood glucose control. This in turn leads to the cardinal features of diabetes mellitus (polyuria, polydipsia and weight loss). Thus, parallel failure of two structurally and functionally complex long-lived cell types with limited capacity for replacement (pancreatic beta cells and podocytes) represent the diabetes-associated nephropathy phenotype. Hyperglycemia itself through oxidant injury or other mechanisms could also potentially contribute to podocyte detachment through various signaling pathways, mTORC1 complex activation via glucose sensors, or direct glucose toxicity effects^7^. However, in the fa/fa rat hyperglycemia itself did not appear to be required either to initiate podocyte injury or to sustain the progression mechanism once it was established. Hence the interaction between hyperglycemia and podocyte loss is represented by dashed lines in the schematic.

Translation of fa/fa rat findings to man has important caveats. First, is the fa/fa rat a model of human obesity-associated nephropathy or type2 DAN or both? Obesity in the absence of overt diabetes is well-known to be associated with glomerular enlargement, proteinuria and development of FSGS lesions^43^. Furthermore, in this setting reducing food intake through bariatric surgery and other approaches ameliorates proteinuria^44^. On the other hand, type2 DAN also results from excessive food intake causing weight gain/obesity and hyperinsulinemia with superimposition of insulin resistance sufficient to cause pancreatic islet beta cell failure resulting in overt diabetes, as also occurs in the fa/fa rat model. Therefore, the fa/fa rat also resembles type2 DAN, particularly with respect to childhood type2 diabetes^45^. A second related question is that although the fa/fa rat replicates morphologic characteristics found in association with type2 DAN including basement membrane thickening, PAS-positive mesangial expansion and high level proteinuria^22^, like most but not all other rodent models, it does not exactly replicate major nodular glomerulosclerosis considered to be a characteristic pathologic phenotype of DAN^23,24^. However, in reality, there is a wide range of pathology observed in biopsies performed on proteinuric diabetics with the proportion of biopsies showing non-classical diabetic features ranging from 14–83%^46^ with 22% having FSGS^47^. The data in this report taken together with previous morphometric studies using a model of growth-induced glomerular failure^12,15^ and model of both type1 and type2 DAN in man^17–19,48–50^, would be consistent with an underlying process initiated by a combination of glomerular enlargement with podocyte susceptibilities. Podocyte susceptibilities could result from the growth factor milieu triggering podocyte mTORC1 activation, hyperglycemia level and exposure time (Fig. 9), metabolic factors including reactive oxygen species, mutations in podocyte-expressed genes such as the *Col4α3* gene recently reported to be associated with MODY^51^, superimposed hypertension and other factors. According to this perspective the fa/fa rat models key elements common to both obesity and type2 diabetes-associated nephropathies. Importantly, these events can be monitored non-invasively using urine pellet podocyte mRNAs from an early stage prior to onset of hyperglycemia or albuminuria.

## Materials and Methods

The study was approved by the University of Miyazaki Animal Research Committee (approval numbers: 2011-539 and 2017-501). Authors confirm that all the experiments are performed in accordance with approved guidelines.

### Rat model of diabetic nephropathy

Male leptin-deficient Zucker (fa/fa) rats that develop type2 diabetes (n = 6) were compared with heterozygous Fa/fa rat littermates (n = 7) as non-diabetic controls. Rats were purchased from Charles River Breeding, Yokohama, Japan at 5weeks old. Open kidney wedge biopsy was performed with removal of about 1/10th of the kidney at 15 and 30 weeks. Kidneys were harvested at 46weeks at euthanasia when kidneys were perfusion-fixed with paraformaldehyde/lysine/periodate (PLP) and paraffin-embedded prior to sectioning.

### Food intake reduction study (calorie reduction, CR)

5 weeks old male fa/fa rats (150 g) were fed ad-libitum until 15weeks of age when they were randomly assigned to either: (i) ad-libitum diet (n = 5), or (ii) 40%CR (n = 6). Food delivered to the 40%CR group was calculated by measuring the food eaten by the ad-libitum group at the same age. Rats were euthanized at 30weeks when kidneys were perfusion-fixed for analysis.

### RNA analysis and qRT-PCR assay

Rats were housed in metabolic cages for overnight timed urine collection (average 17 hr) every 4weeks to measure urine volume, albuminuria, urinary glucose and urinary podocyte mRNA markers (podocin and nephrin) and aquaporin2 mRNA excretion. Urine was centrifuged at 4 °C for 15 min at 3200 × *g* on a tabletop centrifuge. The supernatant was removed, the pellet suspended in 1.5 mL diethyl pyrocarbonate-treated phosphate-buffered saline, and then centrifuged at 12,000 × *g* for 5 min at 4 °C. Diced rat cortex from kidney biopsy samples and washed urine pellets were suspended in RLT/β-mercaptoethanol buffer (RNeasy kit; Qiagen, Germantown, MD, USA) and then frozen at −80 °C^11,16^. RNA from the urinary pellet and kidney cortex were purified using an RNeasy mini kit (cat. No. 74106; Qiagen). cDNAs were reverse-transcribed from total RNA (~1 μg) using a high-capacity cDNA reverse transcription kit (Applied Biosystems, Foster City, CA, USA). Quantitation of podocin, nephrin, aquaporin2 and glyceraldehyde-3-phospate dehydrogenase (GAPDH) mRNA abundance was performed with a LightCycler 96® system (Roche Molecular System, Mannheim, Germany) using FastStart Essential DNA Probe Master Mix (Roche Molecular System, Inc.) in a final volume of 10 μL per reaction. TaqMan probes (Applied Biosystems) used were: rat NPHS1 (nephrin) (cat. No. Rn00575235_m1); rat NPHS2 (podocin) (cat. No. Rn00709834_m1); rat aquaporin 2 (cat. No. Rn00563755_m1); rat GAPDH (cat. No. Rn01775763_m1). cDNA standard curves were constructed using these serially-diluted standards^11,16^. For analysis all urine measurements were adjusted to 24 hours.

### Podometric analysis and phosphor-S6 immunostaining

Immunofluorescence and immunoperoxidase staining used PLP-perfused paraffin-embedded kidney sections. Mean glomerular radius (r) for 30–50 consecutive glomerular profiles was measured moving systematically from outer cortex to juxtamedullary glomeruli using the WinROOF imaging software (Mitani Corp., Tokyo, Japan). Mean maximal R = 4/πr and mean glomerular volume = 4/3πR^3^ were calculated^16,52^. Podocyte nuclear number was measured using Wilms’ Tumor 1 (WT1) immunofluorescence staining using 1.5um thick sections by ImagePro software (Media Cybernetics, Rockville, MD, USA) in 30–50 glomerular tufts^52^. Podocyte nuclear density was estimated using a quadratic equation developed to correct for section thickness and nuclear shape^52^. Average podocyte nuclear number per glomerular tuft = average glomerular volume x podocyte nuclear density. GLEPP1-positive tuft area was estimated from 30–50 consecutive glomerular tuft areas as the %glomerular tuft area staining positive by GLEPP1 immunoperoxidase using the WinROOF imaging software^16,52^. Average total podocyte volume was estimated by multiplying the mean glomerular volume by the mean %GLEPP1-positive area. The non-podocyte glomerular volume was estimated by subtracting total GLEPP1 podocyte volume from glomerular volume. Reagents: WT1 primary antibody (SC-7385 monoclonal IgG1; Santa Cruz Biotechnology, USA) 1:50 with Cy3-labeled secondary antibody (Jackson ImmunoResearch Laboratories, USA) 1:100. Phospho-S6 primary antibody (#2211 polyclonal; Cell Signaling Technology, USA) 1:400 with FITC-labeled secondary antibody (Jackson ImmunoResearch Laboratories, USA) 1:100. Slides were mounted using SlowFade Gold antifade reagent with DAPI (S36939; Invitrogen, USA). Anti-GLEPP1 mouse monoclonal antibody (1B4) at 1:100 (Wiggins laboratory) with immunoperoxidase Vectastain Elite ABC kit (PK-6100; Vector Laboratories Inc, USA).

### WT1 or PAX8/Ki67 double label analysis

In each section cells were characterized as to whether they were podocytes (WT1 positive nuclei) or PECs (PAX8 positive nuclei) according to specific markers and whether they were non-podocyte glomerular tuft cells, periglomerular or interstitial cells or tubular cells according to their position in relation to other cortical structures. Within these compartments cells whose nuclei were Ki67 positive were counted for Fa/fa (6week), fa/fa (6week), Fa/fa (30week), fa/fa (30week), Fa/fa (45week) and fa/fa (45week) groups (n = 5–6). Averaged values for the 12 consecutive images were used for each rat. Thus in the case of WT1 and PAX8 cell nuclei would be double-labelled for WT1/Ki67 or PAX8/Ki67 respectively, or in the case of other compartments would be labelled by Ki67 alone. Double immunofluorescent labeling was performed on 1.5um thick sections using the cell cycle marker Ki67 (Cat # NB600-1252, Novus Biologicals, USA) and either the podocyte nuclear marker WT1 or the parietal cell (PEC) marker PAX8 (Cat # ab53490, Abcom, USA) with DAPI (blue) staining. Compound images from the red, green, and blue channels for 12 consecutive glomerular profiles were made for each rat at 200x magnification with a glomerulus in the center of the field.

### Glomerulosclerosis index

Estimated by a blinded observer using AZAN-stained histologic sections as %glomerulosclerosis for 30–50 consecutive glomeruli.

### Urinary IGF1, IGF2 and serum insulin

24 hr urinary IGF1 and IGF2 excretion were measured using Rat IGF1 and IGF2 ELISA kit (Cat No. MBS824704 and MBS824828, MyBioSource.com, USA). Serum insulin was evaluated using Mercodia Rat Insulin ELISA kit (Mercodia AB, Sweden).

### Statistical analysis

GraphPad PRISM software, version 6.0 (GraphPad Software, Inc., USA) was used. Supplemental Table 1 data are expressed as mean ± SD, other data as mean ± SEM. Differences among two groups were tested using the Mann–Whitney U test, and more than two groups were tested using the Kruskal–Wallis test. When the result of the Kruskal–Wallis test was significant, a Dunn test was performed for *post hoc* analysis. Correlations between parameters were compared using Spearman’s rank correlation coefficient analysis. Correlation P values were calculated using Stata 15 I/C (College Station, TX). A *P*-value < 0.05 was considered statistically significant.

## Supplementary information

Supplemental Information
