# Supplementary material for: Podocyte hypertrophic stress and detachment precedes hyperglycemia or albuminuria in a rat model of obesity and type2 diabetes-associated nephropathy
Source: Sci Rep. 2019 Dec 6;9:18485. doi: 10.1038/s41598-019-54692-z (PMC6898392; doi:10.1038/s41598-019-54692-z)
Supplement: Supplementary file 1 — Supplemental Information [file 41598_2019_54692_MOESM1_ESM.pdf]

Podocyte hypertrophic stress and detachment precedes hyperglycemia or albuminuria in a rat model of obesity and type2 diabetes-associated nephropathy

Akihiro Minakawa[1,3], Akihiro Fukuda[1,2], Yuji Sato[1], Masao Kikuchi[1,3], Kazuo Kitamura[3], Roger C. Wiggins[4], Shouichi Fujimoto[1, 5]

[1]Division of Nephrology, Department of Internal Medicine, Faculty of Medicine, University of Miyazaki, Miyazaki, Japan. [2]Department of Endocrinology, Metabolism, Rheumatology and Nephrology, Faculty of Medicine, Oita University, Yufu, Japan. [3]First Department of Internal Medicine, University of Miyazaki, Miyazaki, Japan. [4]Division of Nephrology, Department of Internal Medicine, University of Michigan, Ann Arbor, Michigan, USA. [5]Department of Hemovascular Medicine and Artificial Organs, University of Miyazaki, Miyazaki, Japan

Corresponding Author: Akihiro Fukuda

Department of Endocrinology, Metabolism, Rheumatology and Nephrology, Faculty of Medicine, Oita University, Yufu, Japan

1-1 Idaigaoka, Hasama-machi, Yufu City, Oita, Japan, 879-5593

Tel: +81-97-586-5793 Fax: +81-97-549-4480

Email: akifukuda@oita-u.ac.jp

A.M. and A.F. contributed equally to this work and are designated coequal first authors.

R.W. and S.F. contributed equally to this work and are designated coequal senior authors.

Supplemental Table 1

Group	Ki67-Positive Nuclei per Field				
	Intra-glomerular	Podocyte	PEC	Periglomerular/ Interstitial	Tubular
**Fa/fa (6week)**	0.7±0.3	0.0±0.0	0.1±0.0	1.2±0.2	1.5±0.3
**fa/fa (6week)**	0.4±0.1	0.0±0.1	0.1±0.0	0.6±0.2 [a]	1.4±0.2
**Fa/fa (30week)**	0.4±0.3	0.0±0.0	0.2±0.1	1.8±0.2	2.2±1.2
**fa/fa (30week)**	0.6±0.3	0.0±0.0	0.2±0.1	2.3±1.8	2.3±1.0
**Fa/fa (45week)**	0.5±0.2	0.0±0.0	0.1±0.2	3.6±0.8 [a]	2.6±0.8
**fa/fa (45week)**	0.9±0.4	0.1±0.1	0.3±0.2	13.9±11.7 [a]	9.1±6.2 [a]

Supplemental Table 1. Ki67 cell cycle analysis: Ki67-positive cell nuclei in different kidney compartments were counted in kidney sections for Fa/fa (6week), fa/fa (6week), Fa/fa (30week), fa/fa (30week), Fa/fa (45week) and fa/fa (45week) groups (n=5–6) shown at left. Statistical indicators using ANOVA with Dunn adjustment compare each group with the Fa/fa (6week) group. There were no statistically significant changes in podocyte and PEC Ki67-positive nuclei in any group. These data show no evidence for cell cycling of podocytes and parietal epithelial cells (PECs) at these time points. In contrast, WT1/Ki67 double-labelled positive cells were observed in 2day old rat S-shaped body developing glomeruli (data not shown). Data are shown as mean±1SD. Fa/fa rats; leptin-deficient homozygous Zucker diabetic Fatty rat, fa/fa rats; leptin-deficient heterozygous Zucker diabetic Fatty rat. [a]: $P<0.05$.

Supplemental Table 2

Quantitative analysis of podometric data from urine and kidney

Normal podocyte loss rate from rat glomeruli and kidneys

Assuming that normal Fa/fa rat glomeruli lose podocytes at the same rate as human glomeruli (0.40%/year) (see Table legend for background data).
Each normal (Fa/fa) rat glomerulus has on average 157 podocytes (see Figure 2b).
Therefore, normal podocyte loss rate = 157x0.40/100 = 0.63 podocytes lost/year
Normal podocyte loss rate = 0.0017 podocytes lost/glomerulus/day.

Two normal rat kidneys contain approximately 70,000 glomeruli (ref 1)
Therefore, under normal conditions rats lose = 0.0017x70,000
= **119 podocytes/day from two rat kidneys under normal conditions**
which can be measured by RT-PCR in the normal rat urine pellet.

Estimated rate of podocytes loss from fa/fa rat glomeruli derived from urine pellet podocin mRNA measurements over the 40week observation period

The urine pellet mRNA signal in fa/fa rats is on average 88-fold higher rate than for Fa/fa control rats over the time course of observation (see Figure 5a).
Therefore, the average rate of podocyte loss from fa/fa rats over the period of observation based on the measured urine podocin mRNA will be 0.0017x88
= **0.15 podocytes lost/glomerulus/day**.

Observed rate of podocyte loss from fa/fa rat glomeruli

The measured slope of podocyte loss from fa/fa glomeruli = -1.49x where x is measured in weeks (see Figure 2b)
Therefore, the observed rate of podocyte loss per day = -1.49/7
= **0.21 podocytes lost/glomerulus/day**

Mechanism of podocyte loss from glomeruli in fa/fa rats

From the above data 71% of podocytes lost from glomeruli of fa/fa rats was detected in the urine pellet over the 40week observation period.

Supplemental Table 2: Comparisons of observed podocyte loss rate from glomeruli to estimated podocyte loss rate derived using the urine podocin mRNA marker in fa/fa rats. In spite of being able to measure podocyte-specific mRNAs in normal rat urine, the normal podocyte loss from rat glomeruli is low. This means that it is difficult to measure the rate of podocyte loss from normal rat glomeruli over the 2year rat life-span where the predicted number of podocytes lost per glomerulus conditions would be expected to be only about 0.8% (i.e. about 1.5 podocytes lost/glomerulus/2years under normal conditions). On the other hand, the longer human life-span (>70years) has allowed this estimate to be made for normal human glomeruli which start life with 580 podocytes per glomerulus and lose podocytes at a rate of 2.3/year = 0.40%/year (ref 1,2,3). Therefore, for the estimations shown above, we have assumed that the normal rate of podocyte loss from rat glomeruli will be proportionally the same as for human glomeruli (i.e. 0.40%/year).

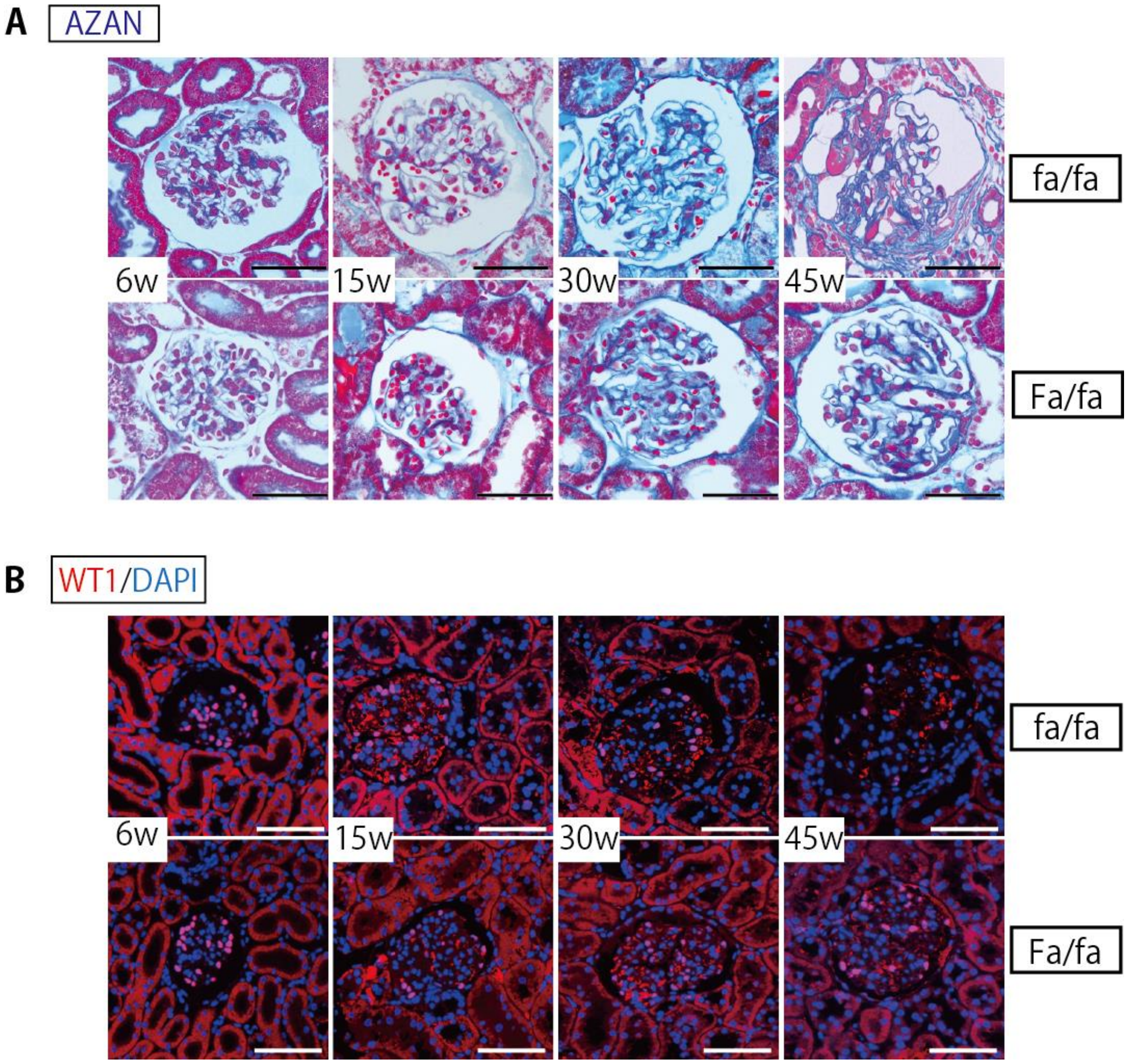

Supplemental Figure 1. Representative histological figures in fa/fa and Fa/fa control rats. (A) Representative AZAN staining at 6, 15, 30 and 46 weeks of fa/fa and Fa/fa rats (bar=50µm). (B) Representative podocyte nuclei identified using Wilms' Tumor 1 (WT1) and WT1/DAPI immunofluorescence staining at 6, 15, 30 and 46 weeks of fa/fa and Fa/fa rats (bar=50µm).

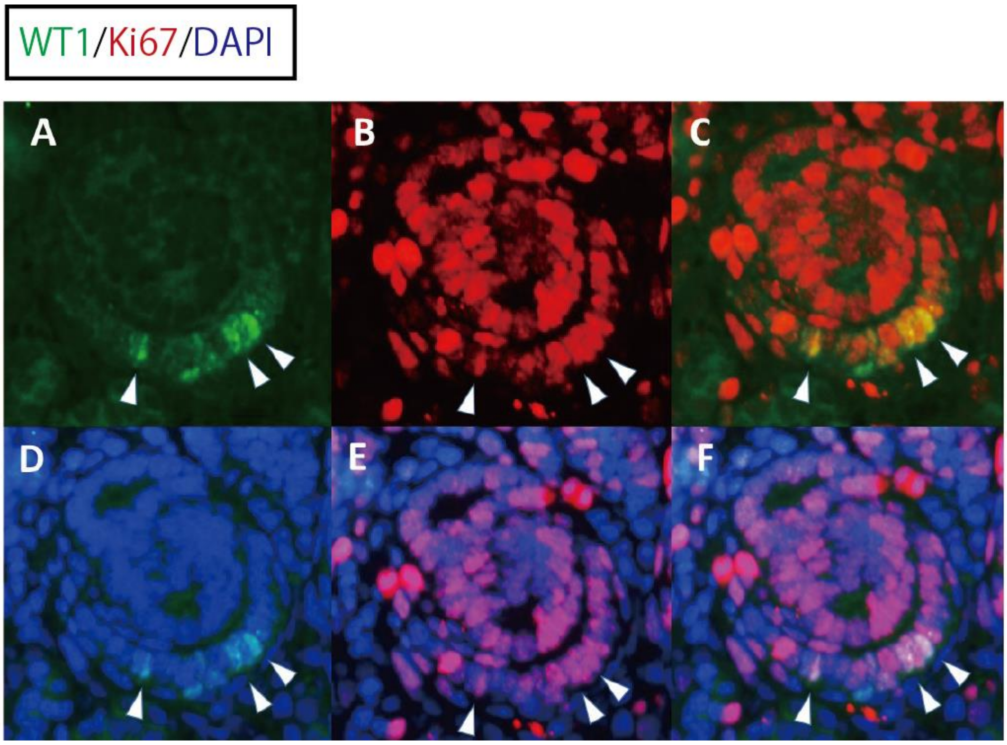

Supplemental Figure 2. 2day old rat kidney showing a developing glomerulus in the S-shaped stage. (A) WT1 immunostaining (green) showing early developing podocytes acquiring WT1 expression. (B) Ki67 staining (red) showing many glomerular cells in the process of cell cycling. (C) Merge of A and B showing that some Ki67 positive cells express WT1 (organge). (D) Merge of A with DAPI showing that the WT1 staining is nuclear (pale green). (E) Merge of B and DAPI showing that Ki67 staining is nuclear. (F) Merge of A, B and DAPI showing that Ki67 positive cycling cells express WT1 in nuclei in developing glomeruli at the S-shaped stage. These data show that dividing cells destined to become podocytes express both nuclear Ki67 and nuclear WT1 at the same time, as also reported in podocyte cell lines for BrdU and WT1 by Mundel and colleagues (ref 4).

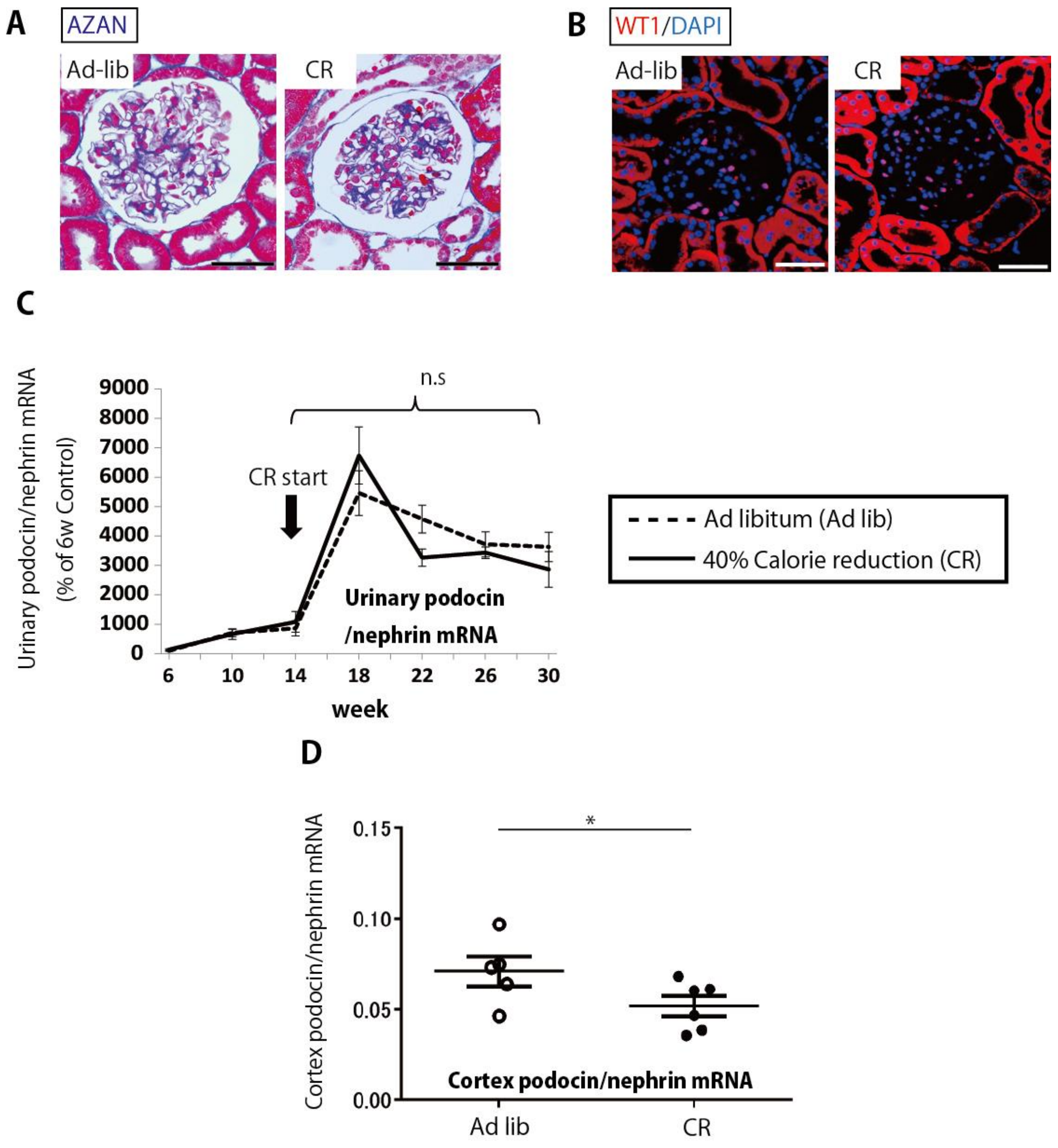

Supplemental Figure 3. Representative histological figures, urinary and kidney cortex mRNA in ad-libitum and CR groups in fa/fa rats. (A) Representative AZAN staining at 30weeks of ad-libitum and 40% calorie intake reduction (CR) group in fa/fa rats (bar=50μm). (B) Representative podocyte nuclei identified using Wilms' Tumor 1 (WT1) and WT1/DAPI immunofluorescence staining at 30weeks of ad-libitum and 40% calorie intake reduction (CR) group in fa/fa rats (bar=50μm). (C) Time course of urinary podocin:nephrin mRNA ratio. (D) Kidney cortex podocin:nephrin mRNA ratio at 30week.

References

1. Heilmann M. *et al*. Quantification of glomerular number and size distribution in normal rat kidneys using magnetic resonance imaging. Nephrol Dial Transplant 27, 100-107, (2012).
2. Hodgin JB. *et al*. Glomerular aging and focal global glomerulosclerosis: A podometric perspective. J Am Soc Nephrol 26, 3162-3178, (2015).
3. Kikuchi M. *et al*. Podocyte number and density changes during early human life. Pediatr Nephrol 32, 823-834, (2017).
4. Mundel P. *et al*. Rearrangements of the cytoskeleton and cell contacts induce process formation during differentiation of conditionally immortalized mouse podocyte cell lines. Exp Cell Res 236, 248-258, (1997).
